# Oral ecological environment modifications by hard-cheese: from pH to microbiome: a prospective cohort study based on 16S rRNA metabarcoding approach

**DOI:** 10.1186/s12967-022-03506-4

**Published:** 2022-07-09

**Authors:** Erna Cecilia Lorenzini, Barbara Lazzari, Gianluca Martino Tartaglia, Giampietro Farronato, Valentina Lanteri, Sara Botti, Filippo Biscarini, Paolo Cozzi, Alessandra Stella

**Affiliations:** 1 Department of Biomedical Science for Health, University of Milan, 20100 Milan, Italy; 2 Institute of Agricultural Biology and Biotechnology, Consiglio Nazionale delle Ricerche (CNR), Via Bassini 15, 20133 Milan, Italy; 3 Department of Biomedical, Surgical and Dental Sciences, School of Dentistry, University of Milan, 20100 Milan, Italy; 4 UOC Maxillo-Facial Surgery and Dentistry. Fondazione IRCCS Cà Granda, Ospedale Maggiore Policlinico, 20100 Milan, Italy; 5 Parco Tecnologico Padano, Loc. Cascina Codazza, Via Einstein, 26900 Lodi, Italy

**Keywords:** pH, Oral microbiota, 16S rRNA metabarcoding, Next generation sequencing, Dental caries, Periodontitis, Cheese

## Abstract

**Background:**

The oral ecosystem conditions dental health, and is known to be positively modified by oral hygiene which cannot always be performed between meals, especially outside home. It is therefore important to identify the practices to be adopted to influence the oral environment in an anticariogenic direction. Milk and cheese are considered functional foods and have a role on oral health. There are several mechanisms by which cheese exerts its beneficial effects on teeth. The aim of the present study was to examine whether short term consumption of hard cheese would affect the oral pH and microbial flora of healthy adults modifying ecological oral environment. The Next Generation Sequencing (NGS) approach was applied to study the effect of Italian Grana Padano (GP), as a prototype of typical hard cheese, on the oral microbiota composition. Finally, we explored *Streptococcus* *mutans*/*sanguinis* ratio as a marker of protective biofilm composition.

**Methods:**

Nine oral-healthy adults were instructed to eat 25 gr of GP cheese for 5 consecutive days. Three time points were chosen for supragingival samples collection and pH measurement. 16S rRNA-gene sequences were obtained both from oral samples and GP cheese using the MiSeq platform and analyzed against the expanded Human Oral Microbiome Database (eHOMD). ProgPerm was used to perform statistical analyses to investigate strain differential representation after cheese consumption.

**Results:**

Taxonomic analyses of the oral microbiota revealed that Firmicutes was the most abundant phylum, followed by Proteobacteria and Actinobacteria. GP cheese significantly modifies oral pH, causing a shift toward basic conditions which are kept for a few hours. The *Streptococcus mutans/Streptococcus sanguinis* ratio lowers in the last observed timepoint.

**Conclusion:**

Our results reveal that a portion of GP cheese eaten after dinner provides important micronutrients (i.e. calcium, vitamins and some aminoacids such as arginine) and changes oral pH toward basic conditions, resulting in a light modification of the oral microbiome towards the reduction of the overall amount of acidophilic bacteria. Furthermore, the *S.* *mutans*/*S. sanguinis* ratio is reduced, contributing to obtain a more protecting environment towards caries establishment and evolution.

**Supplementary Information:**

The online version contains supplementary material available at 10.1186/s12967-022-03506-4.

## Background

Dental caries is the most prevalent noncommunicable disease (NCD), that affects all age groups across the life course all over the world (49) with increasing prevalence in many low-income and middle-income countries [1]. It affects about 95% of the world child population, while periodontal disease, common in the elderly, affects about 15% of the population [2]. Untreated caries in permanent teeth was the most prevalent health condition in 2010, affecting 35% of the global population, or 2.4 billion people worldwide [3]. Changed eating habits, lifestyle and oral hygiene during the COVID-19 pandemic, due to lockdown, wearing mask and the reduced access to dentistry care, increased the risk of developing tooth decay [4].

The human oral cavity is colonized by a microbial community that plays an important role in regulating the oral health status [1, 3, 5–8]. Oral diseases, in particular dental caries and periodontitis, develop as a result of complex perturbations of oral homeostasis. Environmental changes in the oral cavity, including the predominantly acidic or basic pH to which the oral cavity is exposed, affect the microbial community, consequently, it is fundamental to understand the composition of the oral microbial community and the conditions that maintain it in ecological balance to prevent and manage the progression of disease. Food influence is of paramount importance, and dairy foods have long been well known for their role in prevention of dental caries [8]. Several studies have shown anticariogenic properties of milk and cheese [9]. Moreover, recent epidemiological studies in children and adolescents have associated the frequency of milk and cheese consumption to a better oral health. Some of the anticariogenic properties of these dairy foods may be attributable to calcium, phosphate, casein, and postulated mechanisms involve buffering, salivary stimulation, reduction of bacterial adhesion, reduction of enamel demineralization, and/or promotion of remineralization by casein and ionizable Ca, P and casein phosphopeptides [10].

Grana Padano (GP) is one of the most popular Italian hard cheeses. Its typical features are the result of a specific processing technique, a long ripening, and the quality of milk produced by dairy cows fed a specific diet. GP contains a high concentration of proteins and essential aminoacids: leucine, lysine, phenylalanine and tyrosine, to name a few. A very high arginine content makes it a great resource for ammonia producing bacteria, which buffer the oral acid environment. Additionally, being a dairy product, GP supplies proteins and bioactive peptides in a concentrated form. According to CREA (Consiglio per la Ricerca in agricoltura e l'analisi dell'Economia Agraria) tables of food composition (https://www.alimentinutrizione.it/tabelle-nutrizionali/162410), 100 gr of GP PDO contain 1165 mg of calcium and high phosphorus and magnesium concentrations which are important for the development of bone and teeth in children, as well as for their maintenance in all segments of the population. GP is a good source of casein phosphopeptides, known to be essential for teeth remineralization (for nutritional information see Additional file 1: Table S1). Cheese is also an appropriate delivery vehicle for probiotic bacteria [11]. Lastly, a very notable feature of GP as well as other aged hard cheeses resides in the fact that it is lactose-free. Hard cheese consumption is widely spread across the world [12].

Next Generation Sequencing (NGS) showed that oral microbiota is a complex habitat where hundreds or thousands of microbial species co-inhabit the oral cavity and functionally interact [13]. NGS approach has identified more than 700 different predominant bacterial species in the oral cavity, out of which 35% have not been cultivated, yet [14]. However, recently a novel workflow based on targeted phenotypic culturing linked to large-scale whole-genome sequencing, phylogenetic analysis and computational modeling has been described to study the functions and phenotypes of several bacteria believed uncultivable. The application of this model will improve the knowledge about the role of a larger number of bacteria in the whole microbiota community [15]. Several investigations have shown that dental caries is not caused by a single agent, but is associated to an imbalance of microbial species that synergistically cause enamel demineralization by their acidogenic activity [16, 17]. A specific symbiotic relationship between the resident oral microbiota and the host is believed to be essential for maintenance of oral health.

The effect of diet and/or functional food on oral microbiota remains largely unexplored. In the absence of a specific diet, the composition of the salivary microbiota and oral plaque shows no diurnal variation and tends to remain stable for long periods [18, 19]. The aim of this work is therefore to investigate the potential effect of a typical hard cheese consumption on oral health using NGS technologies, by observing relevant changes in microbiome composition across timepoints. We characterized both cheese and oral microbiota to check if cheese could directly transfer its bacteria to the mouth or induce modifications in oral microbiota through other indirect mechanisms.

## Material and methods

### Patients and samples

A prospective cohort study [20] was carried out on nine healthy adult volunteers selected among the residents attending the University of Milan, Department of Biomedical, Surgical and Dental Sciences, Fondazione IRCCS Cà Granda, Ospedale Maggiore Policlinico Milan (Italy), following the STORMS reporting guidelines for human microbiome research [21]. All the subjects were periodontal healthy, according to the International Workshop for a Classification of Periodontal Diseases and Conditions criteria [22], free of caries and without lesions of the mucosa. Exclusion criteria included: diabetes; pregnancy; human immunodeficiency virus (HIV) positivity; use of immunosuppressant medications; antibiotic therapy or oral prophylactic procedures within the past 3 months and during the study; use of probiotics in the period 3 months prior to the study and during it; need for antibiotic coverage before dental treatment; presence of periodontal or gingival diseases; fewer than 28 teeth present in the dentition.

All subjects were instructed to eat a single portion (25 gr) of Italian hard cheese GP after dinner for five consecutive days and to keep a food diary. After the first day (i.e. after the first dinner), participants were asked to abstain from oral hygiene. The next morning, before tooth brushing, they collected a sample of supragingival plaque from the first and second mandibular molar. During the remaining days of the study all subjects brushed regularly their teeth three times per day, using commercially available fluoride toothpaste. All subjects were instructed to refrain from smoking, drinking (except for water) for 8 h, tooth brushing for 3 h and drinking water for 1 h prior to sample collection.

**Fig. 1:**
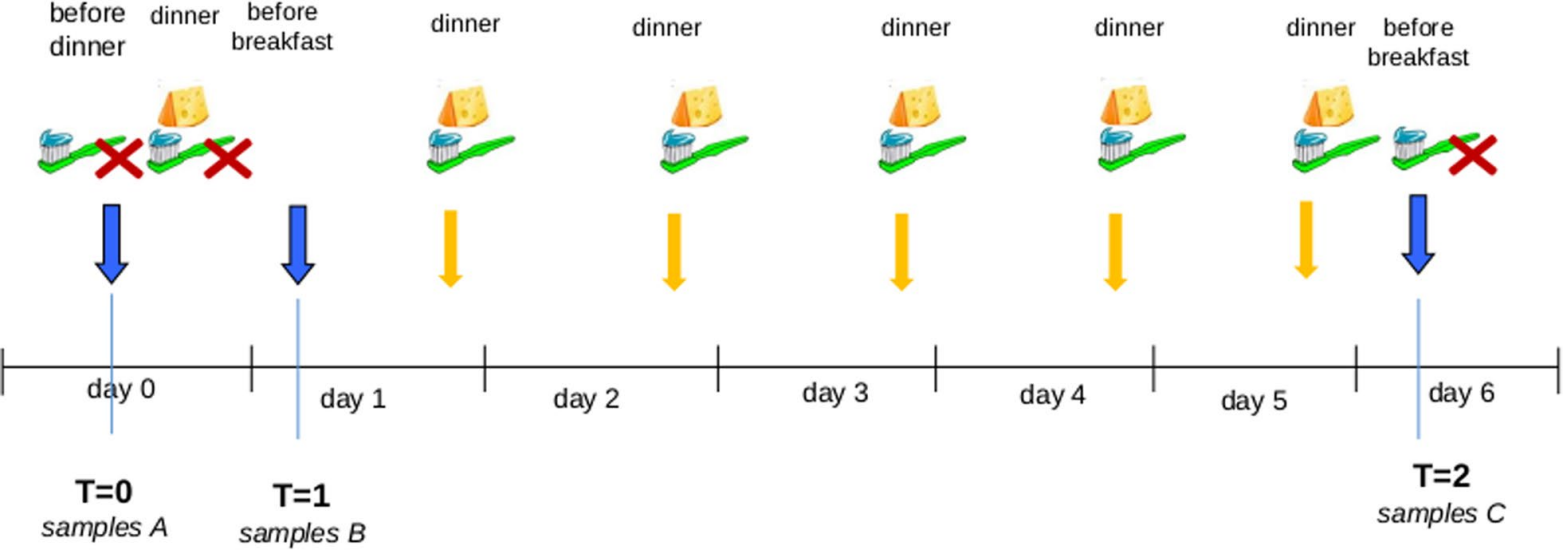
Scheme of sampling. On the top of the picture, the time of the cheese meals (lunch, dinner, etc.) and/or the oral hygiene actions (a red x on the toothbrush means that the teeth were not washed). Yellow arrows indicate the time of the day when cheese was eaten and when the teeth were washed. The Blue arrows indicate the buccal swab sampling collected at T = 0, T = 1 and T = 2. Samples (**A**), (**B**) and (**C**) are, respectively, the samples at different times

Oral samples were collected using the ORAcollect•DNA kit (DNA-Genotek Inc.) according to the kit protocol. After sampling, samples were immediately stored at − 80 °C. Three samples (A, B and C) of supragingival plaque were obtained from each patient at three different times (Fig. 1): Sample A = T0: baseline with no specific pre-treatment; Sample B = T1: the morning after the first consumption of cheese (no teeth brush before sampling); Sample C = T2: the morning after the fifth day of regular consumption of cheese (no teeth brushing before sampling). pH levels were recorded at T0, and after six timepoints (10, 30, 60, 120, 240 and 480 min) after GP cheese assumption.

### Anthropometric analysis and evaluation of food intake

Anthropometric measurements were collected on the nine healthy volunteers, according to the conventional criteria and measuring procedures proposed by Lohman [19]. Body Weight (BW, Kg) was measured by Column scale (Seca 700 balance, Seca Corporation, Hanover, MD, USA) to the nearest 100 g with subjects wearing only light underwear and after bladder emptying, Body Height (BH, cm) was measured to the nearest 0.1 cm using a vertical stadiometer. Body mass index (BMI) was calculated using the formula: BMI = Kg/m2. Volunteers were invited to record their food consumption for five consecutive days using a 24-h food diary to estimate the dietary energy, micro and macronutrients intakes. Full written instructions on how to complete the diary were provided. Diaries were analyzed by specific software that linked diet to a comprehensive database of macro- and micronutrients (Dietosystem, DS Medica, Milan). The estimated daily intake was compared to the dietary reference values for adult standard portions in Italian Diet according to the INRAN guideline (Guidelines for a healthy Italian diet. Italian National Institute for Research on Food and Nutrition) [23].

### pH measurement before and after consumption of a standard portion of hard cheese

After determining the resting salivary pH using pH test strips, the subjects were asked to eat the test food (hard cheese) as previously described.

Salivary pH was measured at time points 0, 10, 30, 60, 120, 240 and 480 min, before eating or drinking or brushing teeth (Saliva-Check BUFFER: D0425-Caries Susceptibility Test-GC America 0.2 resolution).

Unstimulated saliva from each subject, at every single time point, was collected in a plastic disposable container. Immediately after collection, salivary pH was measured using pH strips by dipping the strip into test solution until color development was complete and comparing to the sequence chart on the package to read the pH.

### DNA extraction, library preparation and sequencing

Bacterial DNA was extracted from plaque samples using prepIT®•L2P according to the manufacturer’s instructions. The V3-V4 region of the 16S rRNA was amplified using 16S Metagenomic Sequencing Library Preparation kit (Illumina Inc.). In brief, a PCR amplicon of about 550 bp was obtained with V3 and V4 primer pairs included in the kit. The PCR products were purified with AMPure XP beads and dual indices, and Illumina sequencing adapters were added using the Nextera XT Index Kit. AMPure XP beads were used to clean up the final library before quantification. Nextera libraries were quantified by qPCR using Brilliant III SYBR Green qPCR Master Mix (Agilent Tech.) and the size profile was analyzed on a 2200 TapeStation (Agilent Tech). Libraries were then adjusted to a concentration of 5 µg/ml. Equal volumes of normalized DNAs were then pooled and run on MiSeq (Illumina Inc.).

DNA was extracted from the Italian GP cheese using the kit NucleoSpin soil (Macherey–Nagel). Library preparation and sequencing were carried out as for oral samples.

### Sequence processing and analysis

Raw reads quality was checked using FastQC v0.11.2. Illumina raw sequences were trimmed using Trimmomatic v0.32 with default parameters. Minimum base quality 20 (Phred-scale) over a 4 bases sliding window was required. Only sequences longer than 36 bps were included into downstream analysis. A Qiime formatted taxonomy version of the expanded Human Oral Microbiome database (eHOMD 16S rRNA RefSeq Version 15.22) [24], considering 97% identity to identify cluster, was used to identify the 7 main taxa ranks (superkingdom, phylum, class, order, family, genus, species). For each sample raw data were aligned against the reference database using BWA MEM v0.7. Quantification was performed using SAMTOOLS. The OTU (Operational Taxonomic Unit) table was created using custom scripts. OTU-table in BIOM file format was generated using Qiime Biom convert. Specific primers were built using reference sequences for Rothia dentocariosa, as a proxy for the most relevant species, in order to adjust for relative quantification in the meta barcoding analyses. Prior to quantitative analysis, OTUs from the same genus with unclassified species were grouped, and OTUs with counts in less than one third of the samples were removed.

For oral samples, multidimensional scaling (MDS) was performed with the EdgeR R package (https://bioconductor.org/packages/release/bioc/html/edgeR.html) to explore similarity in microbiota composition in different groups. ProgPerm [25] was used to study both the overall and the individual associations, and to provide measures on the robustness of the discoveries and the reliability of the results. The method progressively permutes the grouping factor labels of microbiome samples and performs differential testing by applying a Wilcoxon rank-sum test to the permuted data in each scenario. The signal strength (− log10 p-values) of top hits from the observed data is compared with their testing performance in permuted data sets to discriminate between significant and nonsignificant hits.

### Arginolytic bacteria identification in the oral microbiome

To identify potentially arginolytic bacteria present in our dataset, we checked for the presence of the arginine deiminase *arcA* (*alias sagP*) gene in organisms included in the AnnoTree database [26] and matched the retrieved bacteria with those present in our dataset.

## Results

### Nutritional status

Five females and four males, with mean age 36 ± 4 years participated in the trial. Their macro- and micronutrients intake was adequate in relation to daily recommendation, apart from calcium below RDA, in agreement with the general population data (Table 1).

**Table 1:** nutritional status. Data are shown as daily mean ± standard deviation

	Total intake	Vs. recommended
Kcal/day	1701.8 ± 461.3	
% Proteins	16 ± 1.89	1
% Lipids	35.6 ± 4.34	8.1
% Carbohydrates	46.32 ± 5.36	− 6.2
Fibers gr.	16.2 ± 4.63	− 8.8
Ca mg	854.4 ± 201.8	− 145.6**
Fe	11 ± 3.2	
Na	2753 ± 761	
K	2342 ± 652	
P	1024 ± 257.7	
Zn	11 ± 5.5	
Folate	240 ± 70.74	
Vit E	6.89 ± 1.37	

**Significant deficit compared to daily recommendations according to the CREA guideline

### Influence of hard cheese assumption on salivary pH levels

**Fig. 2:**
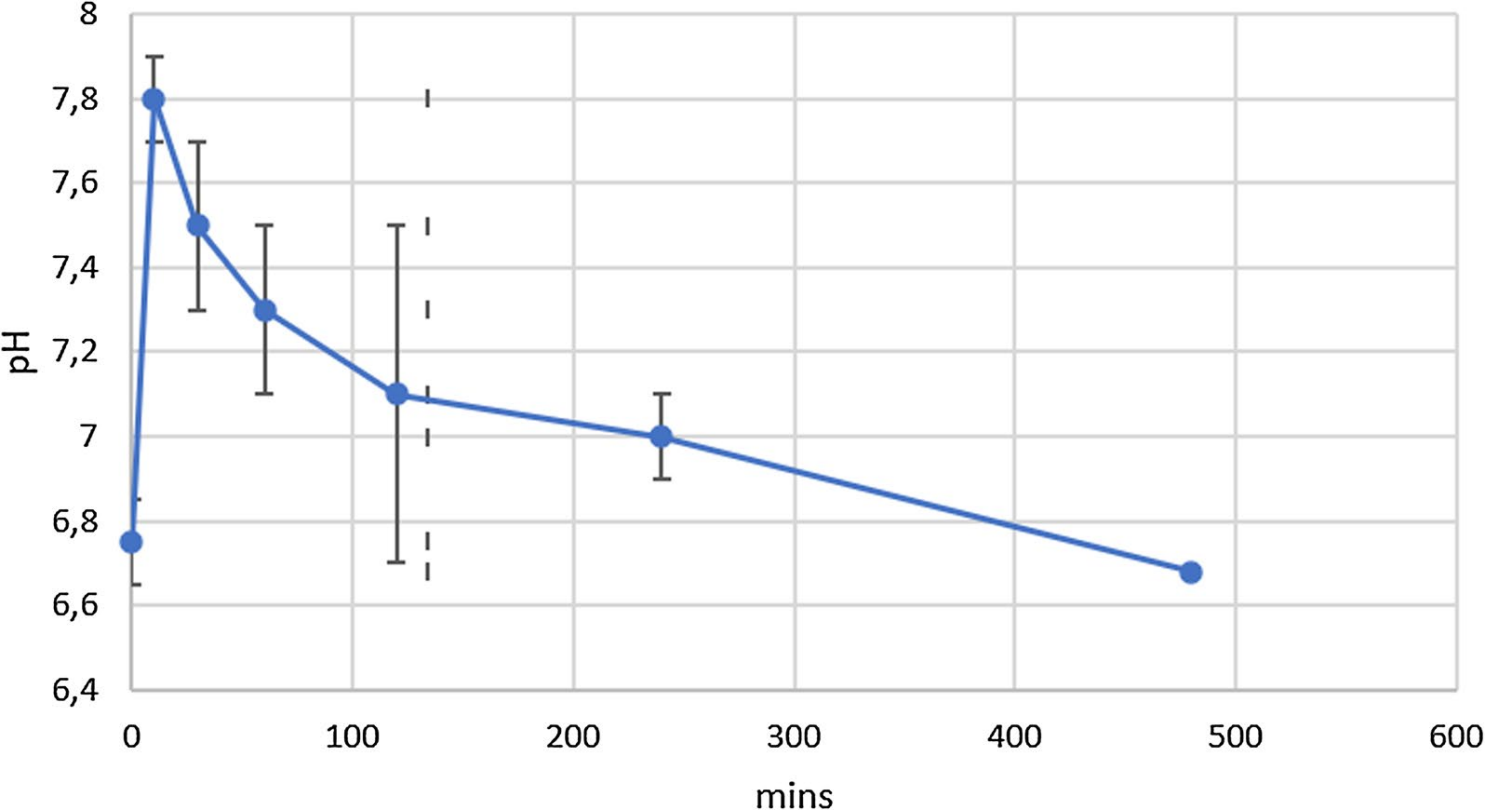
pH measurements at the seven recovered timepoints with standard deviation among samples

pH levels were recovered just before eating a standard portion of GP cheese (T0) and at six time points (10, 30, 60, 120, 240 and 480 min) after GP cheese assumption. In the first 10 min salivary pH showed a significant increase, reaching a peak from baseline. Thereafter, the pH showed a small progressive and slow decline to baseline after more than 4 h (Fig. 2).

### GP cheese microbiota composition

**Fig. 3:**
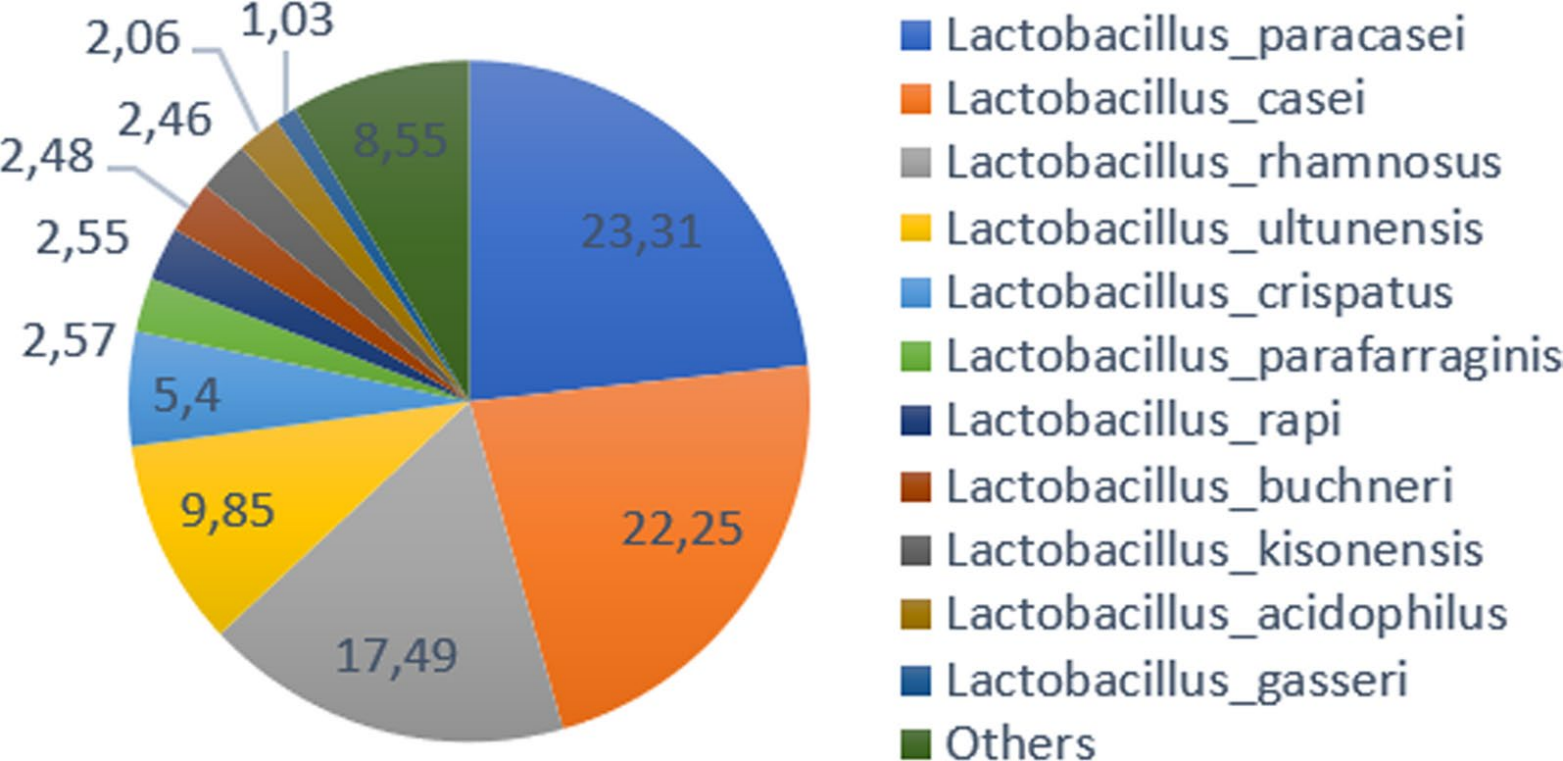
Species composition of GP cheese. Species represented by less than 1% OTUs were grouped

A total of 342,166 reads were obtained from the GP cheese sample, representing 329 OTUs after grouping OTUs from the same genus with unclassified species (Additional file 2: Table S2). Out of these, 126 showed ≥ 10 reads. OTUs with abundance higher than 1% of the total represented OTUs were all Lactobacillus species, Lactobacillus paracasei, casei and rhamnosus accounting for more than 63%, in total (Fig. 3). At the Phylum level, Firmicutes represent the majority of the dataset (96,96%); followed a long way behind by Proteobacteria and Actinobacteria (1,66% and 1,20%, respectively).

### Microbiota profiling in oral samples

**Fig. 4:**
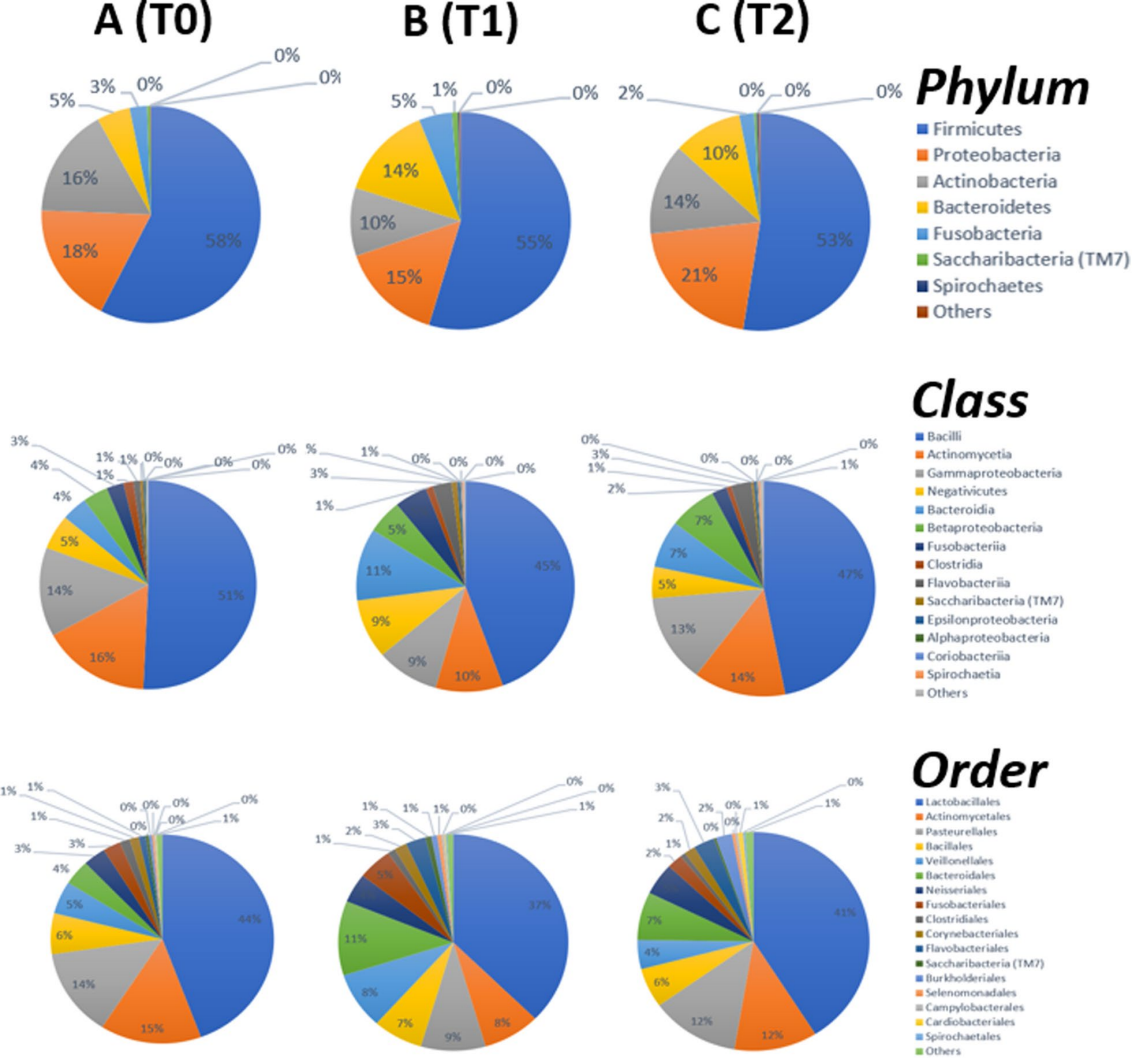
Pie chart showing the percentage of the major Phyla, Class and Order identified in samples **A** (T0), **B** (T1), and **C** (T2) by 16S rRNA analysis

A mean of 758,441 reads per sample (standard deviation 213,584, min 131,797, max 1,279,983) was obtained. eHOMD version 15.22 contains 1015 OTUs, 535 of which were recovered in this investigation, after grouping OTUs from the same genus with unclassified species (Additional file 3: Table S3). The percentage of different phyla, classes and orders in groups A, B and C is reported in Fig. 4.

The most abundant phylum detected in all groups is Firmicutes (58%, 55%, 53% in A, B, and C, respectively), followed by Proteobacteria, Actinobacteria, Bacteroidetes, and Fusobacteria. Bacilli are the most abundant class (51% in A, 45% in B, and 47% in C), followed by Actinobacteria and Gammaproteobacteria. Lactobacillales is the most abundant order (44% in A, 37% in B, and 41% in C). The number of OTU in the full lineage at each timepoint is reported in Additional file 4: Table S4. As a control, specific primers for *Rothia dentocariosa*, one of the most represented species in all datasets, were used to validate the numeric representation of reads, confirming data obtained with the bioinformatic procedure leading to read counts.

**Fig. 5:**
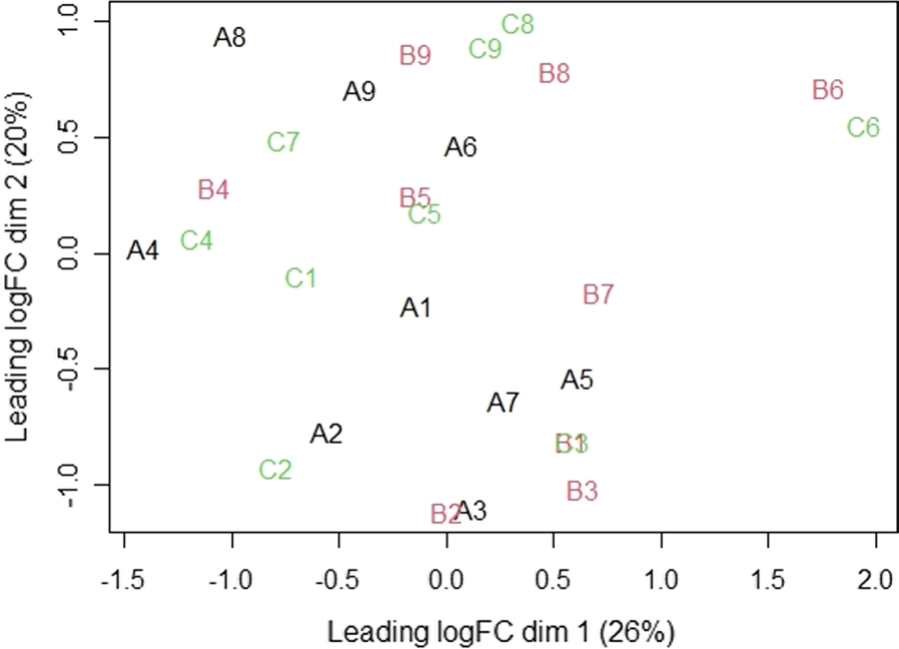
Multidimensional Scaling (MDS) plot of microbiota of different samples taken at different time points **A** (black), **B** (red) and **C** (green)

The MDS Plot based on the distribution of all species (Fig. 5) shows that microbiota composition reflects a high heterogeneity across individuals. In fact, samples collected from a same person at the three time points cluster much better than samples from different individuals collected at the same time point.

### Differential abundance analysis

Differential abundance analysis on microbiome data is challenging due to many factors, the main of which are clustering sequence reads into OTUs and data heterogeneity across subjects. The degree of heterogeneity across samples can lead to the false conclusion that the microbiome is not associated with an experimental condition, or that some species are differentially represented across biological datasets, as the effects of the applied condition are masked among differences due to the personal microbiome composition, which is highly variable. This is particularly notable when experimental conditions interfere with quantitatively small effects on microbiome composition, as is the case of our tests. It is thus fundamental to explore both the overall and the individual associations to highlight significant differences between groups of subjects who underwent the same treatment, and to apply statistical pipelines that test the robustness of the highlighted associations.

To analyze our data, we chose to apply the Qiime pipeline for sequence clustering according to OTUs collected into eHOMD v15.22, the most updated and specific repository for oral microbiome representation, and to use ProgPerm for data abundance analysis. This method performs progressive permutation and applies correlation tests to highlight significant differences in OTU representation between experimental conditions. We performed comparisons between groups using all possible combinations, *i.e.* T0 vs T1, T0 vs T2, and T1 vs T2. 530 out of the 535 OTUs recovered after sequence clustering passed the cutoff of exhibiting counts in at least one third of the samples and were analyzed to retrieve abundance differences.

**Fig. 6:**
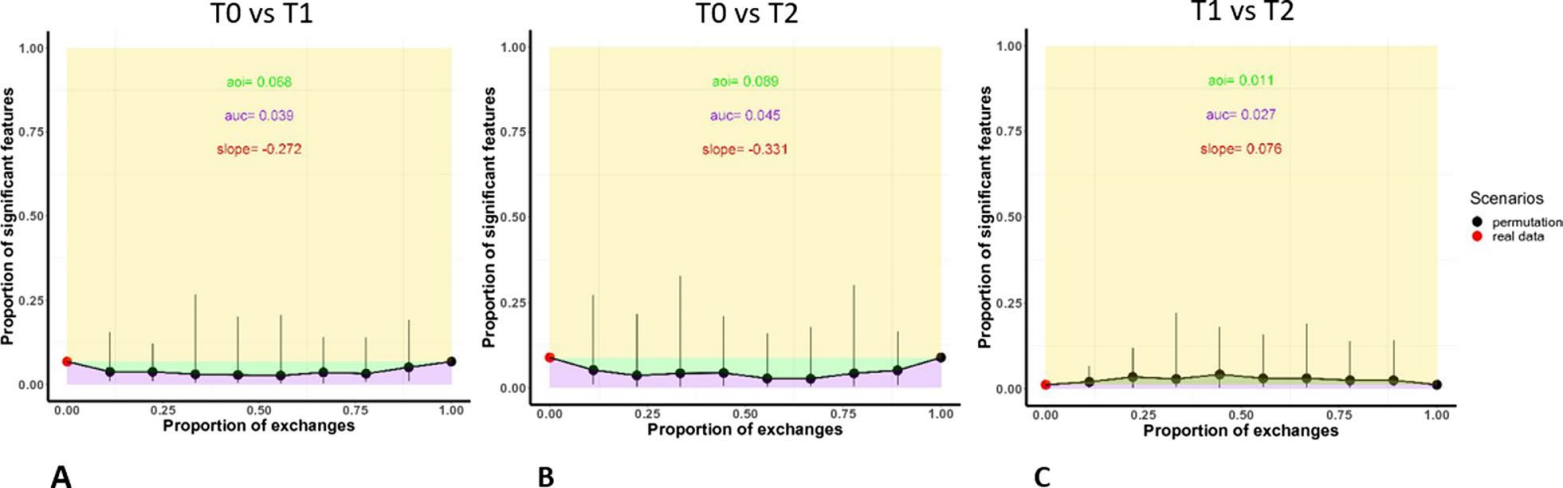
ProgPerm U-Curve of proportion of significant features versus proportion of mixing of the T0 vs T1 (**A**), T0 vs T2 (**B**), and T1 vs T2 (**C**) comparisons. The x-axis describes the proportion of mixing the two groups of data. The y-axis describes the proportion of significant features. The red dot describes the observed data, black dots describe the permuted data. The vertical bars describe the 95% quantile confidence intervals

The T0 vs T1 comparison is aimed at individuating short term microbiome modifications induced by GP cheese consumption. The U-Curve obtained with ProgPerm (Fig. 6A), which can be used as a global measure to depict the overall association between microbiome compositions and different experimental outcomes, shows a light association in the observed data, suggesting a moderate influence of GP cheese eating on oral microbiome composition. This was expected, as we’re observing the effects of a single assumption, which cannot be compared to the effects of more influential long-term treatments that recover differences due to microbiome composition recalibration after long periods of administration of a particular diet, therapy, or drug. In such a condition, the variable’s effect might be modified by other variables and distorted by potential systematic bias, thus, highlighting significant differences between timepoints becomes particularly challenging. A similar behavior can be observed for the T0 vs T2 comparison (Fig. 6B), which aims at studying a more extended effect of eating cheese on the oral microbiota composition (5 days of administration). The T1 vs T2 comparison (Fig. 6C) aims at highlighting differences in microbiome composition due to the effects of GP extended assumption, starting from a condition where GP assumption has already imposed its modifications to the native microbiome composition. In this comparison, the U-Curve denotes the absence of significant variations in microbiome composition between the two datasets.

**Fig. 7:**
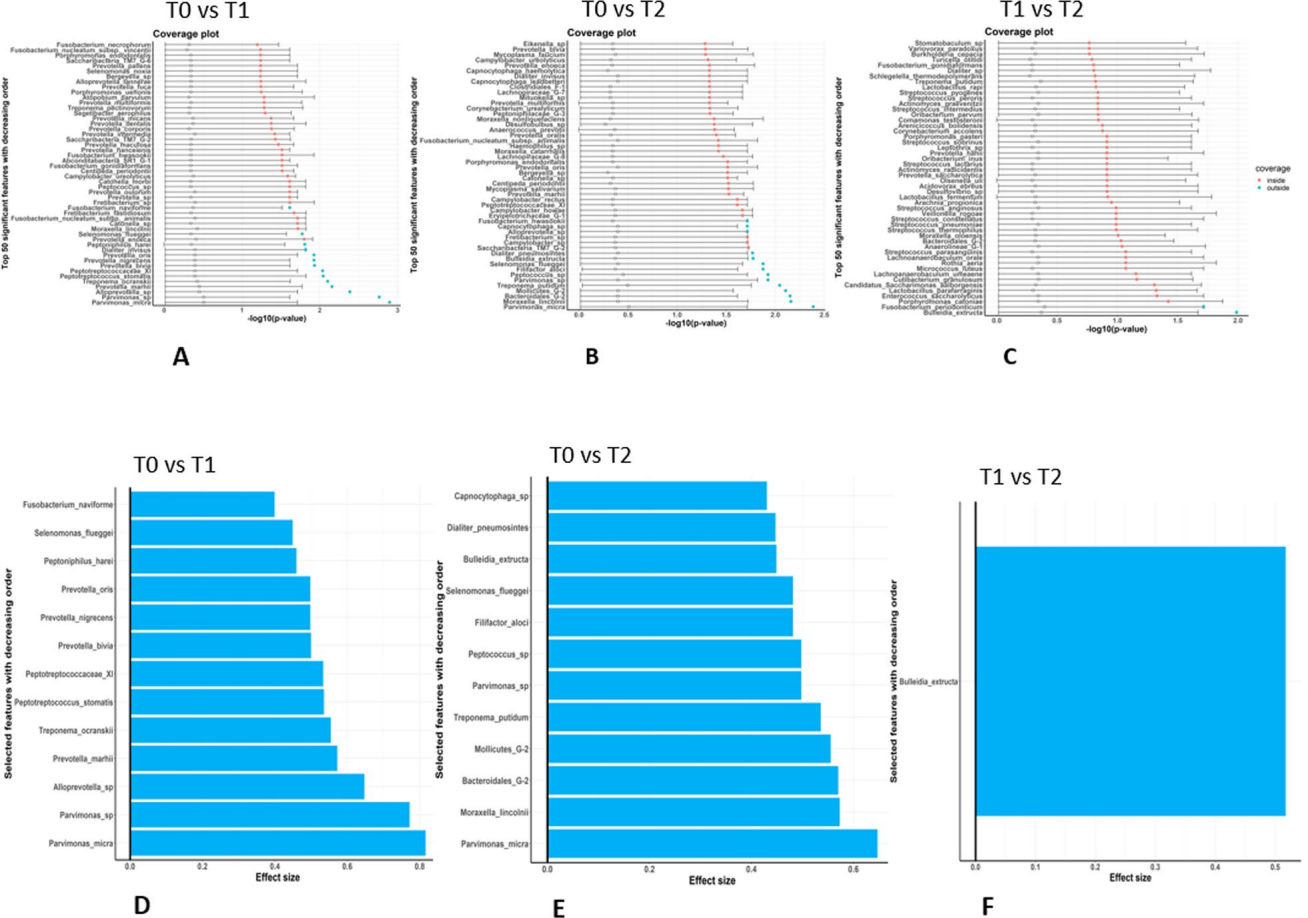
**A**–**C:** P-value coverage plot of the top 50 features in the T0 vs T1, T0 vs T2, and T1 vs T2 comparisons with decreasing order. The color dots denote the − log10 p-value of top 50 features in the original data (permutation proportion is 0). The horizontal bars describe the 95% quantile confidence intervals of the − log10 p-value in the full permutation scenario. **D**–**F** Effect size of the identified features in the T0 vs T1, T0 vs T2, and T1 vs T2 comparisons

Despite the low association denoted by the U-Curves, OTUs with significant differential abundance were individuated in all three comparisons. ProgPerm achieves the identification of robust variables by observing whether the − log10 p-values of targeted features lie within the 95% confidence interval of median − log10 p-values of the full permutation scenario. According to this classification, the T0 vs T1 comparison shows 13 species which are significantly more represented in T1 (Fig. 7A). 12 hits are more represented in T2 within the T0 vs T2 comparison (Fig. 7B). Linear discriminant analysis effect size (LEfSe)—determining the features most likely to explain differences between classes by coupling standard tests for statistical significance with additional tests encoding biological consistency and effect relevance—is also computed by ProgPerm (Fig. 7D and E). In both comparisons, Parvimonas micra—an anaerobic coccus frequently isolated from dental plaque especially in patients with chronic periodontitis, known to harbour aminopeptidase activity—is the species showing the highest effect size, and Parvimonas sp. and Selenomonas flueggei are also recovered. These three bacteria exhibit a significant increment in representation moving from T0 to T1, and T2 levels are very similar to T1 ones. Among the other statistically significant variations, those related to the Prevotella genus, classified as a proteolytic/amino acid-degrading bacteria, are evident in the T0 vs T1 comparison, with four species (marshii, bivia, nigrescens and oris), as well as Alloprevotella sp., overrepresented in T1. Apart from the already mentioned organisms, species recovered in the T0 vs T2 comparison differ from those recovered in T0 vs T1, with Bulleidia extructa—another anaerobic bacterium associated with oral infections, mainly periodontal disease [27]—exhibiting differential representation between the two timepoints, being more represented in T2. Bulleidia extructa is the only species with a significant effect size in the T1 vs T2 comparison (Fig. 7C and F), thus showing that its increment in representation occurs between T1 and T2, after a more prolonged assumption of GP cheese. In none of the three comparisons species being more represented in the former of the timepoints are recovered, and no association is displayed for classical saccharolytic bacteria, such as Streptococcus, Actinomyces, and Lactobacillus species, known to promote dental caries.

### Trend of caries- or periodontitis-related bacteria across timepoints

**Fig. 8:**
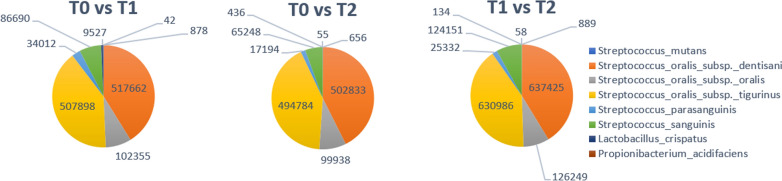
Quantification of known cariogenic bacteria within timepoints

Despite not having been individuated by ProgPerm as statistically significant with respect to their quantitative representation across timepoints, the relative abundance of known cariogenic bacteria such as *Streptococcus oralis, Streptococcus mutans, Streptococcus sanguinis, Streptococcus parasanguinis, Lactobacillus crispatus* and *Propionibacterium acidifaciens* was observed in the three sample collections (Fig. 8). None of these bacteria exhibits a defined trend across timepoints, not even assuming less stringent cutoffs for statistical significance: the only observable differences are mainly due to samples heterogeneity within timepoints (see Additional file 3: Table S3). In all timepoints, *Streptococcus oralis* subsp. *dentisani* and subsp. *tigurinus* are highly represented, followed by *Streptococcus oralis* subsp. *oralis* and *Streptococcus sanguinis*.

**Fig. 9:**
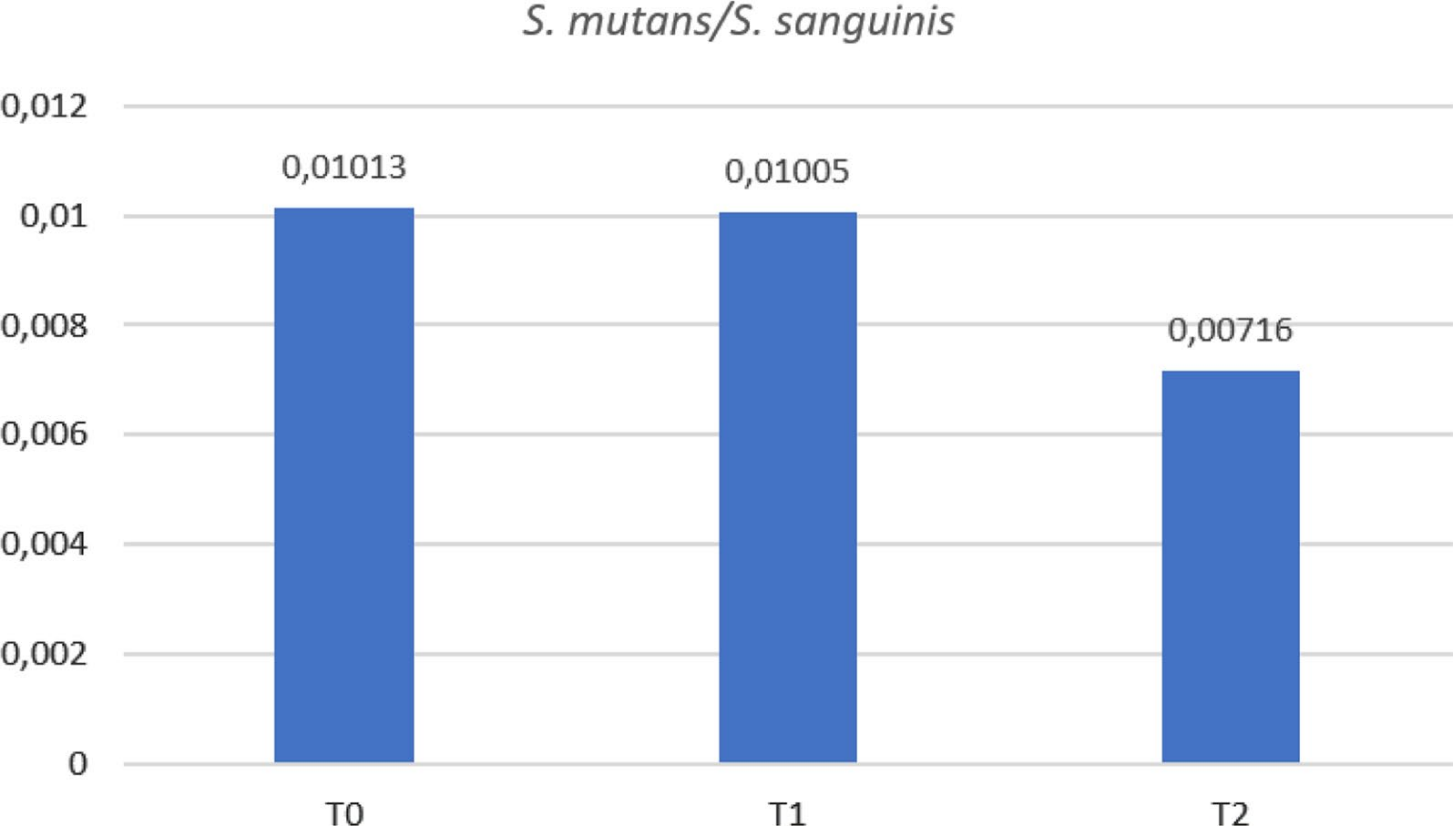
*Streptococcus mutans/Streptococcus sanguinis* ratio at the three timepoints

*Streptococcus mutans* has a demonstrated positive association with the establishment of severe early childhood caries (S-ECC) [28]. *Streptococcus sanguinis* was proposed to play an antagonistic role against *S. mutans* colonization in the oral cavity, thus reducing the risk of caries establishment [29, 30]. The two bacteria representation within the oral flora is extremely different, being *S. sanguinis* far more represented than *S. mutans*. This hampers the interpretation of the effects of their representation with ordinary data analysis techniques, but the observation of their ratio in different clinical situations showed that lower ratios are associated with a lower incidence of dental caries, suggesting that this ratio could be one of the indicators prone to be used to predict caries establishment and evolution in children [30]. In our study, this ratio doesn’t undergo significant changes from T0 to T1, while T2 exhibits a lower ratio with respect to the former timepoints (Fig. 9).

The trend of relative abundance of periodontal bacteria such as *Porphyromonas_gingivalis, Aggregatibacter actinomycetemcomitans, Tannerella forsythia* and different strains of *Treponema denticola* was also verified across timepoints (see Additional file 3: Table S3). As for cariogenic bacteria, the relative abundance of these strains was not statistically significant, but the trend shows that all of them tend to be more represented from T0 to T1 and then to T2, except for *Aggregatibacter actinomycetemcomitans*, which has its lowest representation in T1.

### Behaviour of arginolytic bacteria across timepoints

**Fig. 10:**
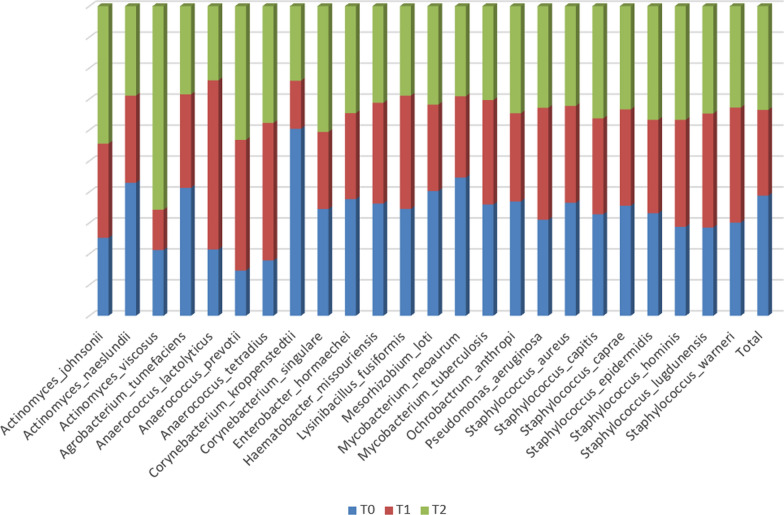
Proportional representation of arginolytic bacteria in the T0, T1 and T2 datasets. Representation in the three datasets was normalized according to the total number of sequences obtained for each timepoint

Being GP an arginine-rich substrate, we investigated the hypothesis that arginolytic bacteria present in the oral cavity may be influenced in their representation by its consumption. In our dataset, we found 24 species having the *arcA* gene, of which proportional representation in all samples, at the three timepoints, is displayed in Fig. 10. Unfortunately, we do not dispose of expression data, and this prevents making any assumption on the activity of the *arcA* gene of these bacteria at the different timepoints. No common trend can be evidenced for arginolytic bacteria as a response to increased arginine availability. None of them exhibits statistically significant differences in representation across timepoints.

## Discussion

Oral microbiota and pH have an important role on dental health. Hence, an appealing hypothesis is that some foods may change the oral microbiota composition favoring the presence of bacteria with a positive effect on oral health. The anticariogenic effect of some food, such as hard cheese, and their ability to modify oral microbiota was not previously studied especially by using a 16S rRNA metabarcoding approach.

The present study focuses on the exploration of GP cheese-dependent short term (1 and 5 days after eating cheese) variation of the oral environment both in terms of pH and microbiota. To extrapolate the effects of presence of cheese microbiota versus the ability of cheese to induce modification of the oral microbiota, microbiota composition of the GP cheese was also characterized by NGS. At a taxonomic level, the percentage of different supragingival plaque bacteria identified in this study before (T0) and after GP consumption (T1 and T2) agrees with previous studies [31–33]. There were small differences at phylum, class and order level in T0 vs T1 and T2, indicating that eating cheese does not cause a big shift in the oral microbiota in a relatively short time span. Several reports showed stable oral microbiome with little or no variation in profiles within subjects over time [19, 32–34]. The stability of the microbiota composition is indispensable to study the effect of functional food on oral microbiota variation.

In our datasets, *Streptococcus oralis* subsp. *dentisani* and subsp. *tigurinus* are the most abundant OTUs in T0, T1 and T2. *Streptococcus sp*., *Haemophilus_parainfluenzae*, *Rothia_dentocariosa*, *Gemella_haemolysans*, and *Streptococcus_infantis* are also recovered within the 10 most represented OTUs at all the three timepoints, while *Rothia aeria* and *Veillonella dispar* are present within the top 10 most represented OTUs only in two out of three datasets (T0 and T2 the former, and T1 and T2 the latter). *Lactobacillus paracasei*, *casei*, *rhamnosus* and *ultunensis*, which represent almost 73% of the total OTUs detected in GP cheese, do not increase their representation moving from T0 to T2, suggesting that no direct bacteria transfer from cheese to the oral microbiota can be postulated.

Other abundant OTUs within the top 30 belong to the *Streptococcus* genus. In healthy individuals, *Streptococci* constitute more than 50% of the oral microbiota [35]. These bacteria generally possess low pathogenic potential. *Streptococci* such as *Streptococcus sanguinis* seem to have a positive effect on oral health: *S. sanguinis* is often found in subgingival biofilm that includes periodonto-pathogens and is correlated with a delay in colonization by these deleterious bacteria [36]. *Streptococcus mutans* is known as a cariogenic microorganism with the ability to colonize teeth, to cause a marked reduction in pH in the presence of a sugar substrate and consequently induce caries [37]. The ratio between *S. mutans/S. sanguinis* has been suggested to be helpful in determining caries risk. A low ratio would imply a low risk of caries and conversely, a high ratio would indicate high caries risk [28–30]. In this study few OTUs of *Streptococcus mutans* were detected, this is expected as this organism is always present in a limited proportion in oral samples, furthermore, all participants to the study were cavities' free. No conserved trend was observed among samples for *Streptococcus mutans* representation during timepoints, suggesting that GP assumption doesn’t influence its participation to the oral microbiome, nonetheless, the *S. mutans/S. sanguinis* ratio decreases in T2 when compared to T0 and T1, suggesting the establishment of a more protective environment with respect to caries development. In our experimental trial *Parvimonas micra* (*P. micra*) shows the highest effect size both in T0 *vs* T1 and T0 *vs* T2, due to a significant increase in representation from T0 to T1, while T1 and T2 show comparable levels. *P. micra* is normally found in the oral cavity and is one of the bacterial species most frequently isolated from infected root canals of teeth with chronic apical periodontitis.

A previous study showed a disparity between microbial communities localized in acidic versus neutral pH strata in dentinal caries [38]. Acidic conditions were associated with low diversity microbial populations, dominated by *Lactobacillus* species*.* In our study, 25 *Lactobacillus* species are recovered, which do not show a common trend during timepoints, being some of them incremented and others reduced moving from T0 to T2. Nonetheless, the total amount of *Lactobacillus* decreases during timepoints, passing from 0.266% of the total sequences mapped to known OTUs in T0, to 0.064% in T1 and 0.042% in T2, suggesting a general effect of the increase of pH due to GP cheese assumption on acidophilic bacteria. In our study, almost all *Prevotella* and *Megasphaera* species increased their representation after GP cheese assumption mostly in T1. *Treponema* species maintained this trend up to T2. *Prevotella* gram negative species were associated to dental caries [39], and some *Prevotella* spp. such as *Prevotella intermedia, Prevotella nigrescens* and *Prevotella melaninogenica* predominate in periodontal disease and periodontal abscesses [40]. However, *Prevotella intermedia* was also associated to oral health [31]. *Treponema sp* was differentially detected in healthy controls *vs* patient cases [36]. Moreover, *Treponema* was associated with dental health, whereas *Lactobacillus*, *Streptococcus* and *Megasphaera* to dentin lesions [41]. On the contrary, in other studies *Treponema denticola* was associated with periodontal diseases such as early-onset periodontitis, necrotizing ulcerative gingivitis, and acute pericoronitis [38]. *Megasphaera* was less frequently found in the caries group [42].

The overall information available on caries establishment and progression, as well as susceptibility to periodontitis, clearly shows that both pathologies cannot be ascribed to a few well identified pathogens, but depend on the interaction of multiple factors and organisms. Oral microbiota is a mirror of the state of health of an individual, and as it can be strongly affected by diet, it is important to determine which foods can be of help in maintaining a balanced and healthy composition. Much attention has been focused on the production of organic acids by oral pathogenic bacteria through the metabolism of carbohydrates. Emerging evidence indicates that alkali generation by bacteria, particularly through ammonia production from arginine and urea, plays a major role in pH homeostasis of oral biofilms and may moderate the initiation and progression of dental caries. Dental caries occurs when the acidification phases outweigh the alkalization phases, hence allowing the establishment of a more acidogenic, less alkalinogenic flora, which in turn results in lower plaque pH values with enhanced and prolonged enamel demineralization. As demonstrated by Stephan’s Curve [43], described by Robert Stephan in 1943 and reflecting plaque pH change following intake of fermentable carboydrates, acid liquids, or sugars in presence of acidogenic bacteria, sugar assumption causes a fall of the pH level under the threshold of 5.5, defined as critical because corresponding to the condition in which the demineralization of the enamel takes place. To reduce decalcification of tooth surfaces, patients should be encouraged to consume foods as hard cheese that do not result in a drop in plaque pH. A very high arginine content makes GP cheese a great source for ammonia producing bacteria which can buffer the oral acid environment. The pH curve shown in this study, in fact, has an opposite trend with respect to Stephan’s curve, showing a significant pH increase in the short term. Additionally, being a dairy product, GP supplies proteins and bioactive peptides as milk does, but in a concentrated form.

## Conclusions

This study examines, for the first time by NGS technology, the short-time effects of eating a portion of hard cheese on the oral microbiome composition and the associated pH variations. NGS analysis of the bacterial composition of GP cheese, conducted in parallel, reveals that there is no direct transfer of microorganisms from cheese to mouth, and that other molecular mechanisms are responsible for microbiota modifications. Even if no direct evidence of a significant modification in the representation of bacteria known to be cariogenic or directly related to periodontitis can be provided, small changes in the amounts of acidophilic bacteria on their whole was observed, together with a decrease in the *S. Mutans*/*S. sanguinis* ratio, which mainly occurs as an effect of eating GP routinely. Although these trends require to be further investigated, they suggest a positive effect on the overall composition of the oral flora towards a healthy status which can lead to a major resistance to caries establishment. Moreover, we have observed that the variations in the alkaline sense that occur in the mouth immediately after the consumption of cheese are maintained over time, up to over four hours. The alkaline environment, together with calcium, phosphorus, caseinphosphopeptides and arginine, contained in large quantities in this cheese, not only inhibits demineralization, but also promotes the process of remineralization, making this food ideal for determining an oral environment that hinders the formation of caries in the periods of time between one meal and another, especially in the conditions of impossibility of correct oral hygiene practices.

## Supplementary Information

**Additional file 1: Table S1.** GP cheese nutritional information.

**Additional file 2: Table S2.** GP cheese OTU classification. The table reports the counts for each OTU found in the GP cheese sample. OTUs from the same genus with unclassified species were grouped.

**Additional file 3: Table S3.** OTU counts in all samples. Total counts for all samples A (T0), B (T1) and C (T2) are given, together with counts of single samples. OTUs from the same genus with unclassified species were grouped.

**Additional file 4: Table S4.** Full lineage of the OTUs recovered in this study. Counts for each species are given as a total for all samples at each timepoint. OTUs from the same genus with unclassified species were grouped.

## Data Availability

The datasets generated and analysed during the current study are available in the ENA Metagenomics repository, Project PRJEB51725, Samples ERS11067207- ERS11067233 (oral samples), and ERS11068245 (GP cheese).

## Abbreviations

GP: Grana Padano
NGS: Next Generation Sequencing
OTU: Operational Taxonomic Unit
