# Supplementary material for: Oral ecological environment modifications by hard-cheese: from pH to microbiome: a prospective cohort study based on 16S rRNA metabarcoding approach
Source: J Transl Med. 2022 Jul 9;20:312. doi: 10.1186/s12967-022-03506-4 (PMC9271248; doi:10.1186/s12967-022-03506-4)
Supplement: Supplementary file 1 — Additional file 1: Table S1. GP cheese nutritional information. [file 12967_2022_3506_MOESM1_ESM.pdf]

Additional Table 1

GP Cheese NUTRITIONAL INFORMATION

Average nutritional values	Per 100 gr	% RI (*) per 100 g
Energy	1597 kJ / 384 kcal	19
Fat	28 gr	40
- of which saturates	18 gr	90
Carbohydrate	0 gr	0
Fibre	0 gr	
Protein	33 gr	66
Salt	1,8 gr	29
Vitamin A	224 µg (28% NRV**)	
Riboflavin	0,36 mg (26% NRV**)	
Vitamin B12	3 µg (120% NRV**)	
Calcium	1165 mg (146% NRV**)	
Phosphorus	692 mg (99% NRV**)	
Magnesium	63 mg (17% NRV**)	
Zinc	11 mg (110% NRV**)	
Copper	0,5 mg (50% NRV**)	
Selenium	12 µg (22% NRV**)	
Iodine	35,5 µg (24% NRV**)	
Arginine	820 mg	

(*) Reference Intake (RI) of an average adult (8 400 kJ/ 2 000 kcal)

(**) Nutrient Reference Value
